# Serial changes in diffusion tensor imaging metrics and therapeutic effects of repetitive transcranial magnetic stimulation in post-traumatic headache and depression: A case report

**DOI:** 10.1097/MD.0000000000037139

**Published:** 2024-03-29

**Authors:** Young-Ji Yun, Gi-Wook Kim

**Affiliations:** a Department of Physical Medicine and Rehabilitation, Jeonbuk National University Medical School, Jeonju, Republic of Korea; b Research Institute of Clinical Medicine of Jeonbuk National University – Biomedical Research Institute of Jeonbuk National University Hospital, Jeonju, Republic of Korea.

**Keywords:** axonal injury, axonal regeneration, diffusion tensor imaging, therapeutic approaches for the treatment of CNS injury, traumatic brain injury

## Abstract

**Background:**

Mild traumatic brain injury patients commonly complain headache and central pain, and the pain accompanies depressive mood change. This case study reports the therapeutic effect of repetitive transcranial magnetic stimulation (rTMS) in mild traumatic brain injury patient with headache and depression through objective serial changes of diffusion tensor imaging (DTI).

**Methods:**

The 51-year-old man complained of headache and depression despite conventional treatment for 13 months. We applied 15 times rTMS on the left dorsolateral prefrontal cortex. We checked the pain and depression through numeric rating scale (NRS) and Beck depression inventory (BDI) when admission, discharged, and 1 month after discharge. DTI was performed 3 times; before, during-day of rTMS 6th stimulation, and after-day of rTMS 15th stimulation. Then the reconstructed White matter related to pain and depression was obtained.

**Results:**

NRS and BDI showed significant improvement and it was maintained 1 year after discharge. DTI-based metrics of the White matters related to pain and depression gradually increased before – during – after rTMS.

**Conclusion:**

Studies focused on examining changes in pain, depression and DTI-based metrics of White matter are rare. This case is significant in that not only pain and depression improved after the rTMS, but also serial changes in White matter were observed in DTI.

## 1. Introduction

Post-traumatic headache (PTH) has a higher prevalence in mild TBI (75.3%).^[1]^ TBI patients also have a 10% to 77% prevalence of depressive and anxiety disorder that may be the most common and disabling psychiatric condition.^[2,3]^ In addition, post-trauma central pain also has a high prevalence range from 22% to 95% for mild TBI.^[4]^ These symptoms of post-traumatic brain injury can greatly affect the daily life of patients.

Medications such as non-steroidal anti-inflammatory drugs, acetaminophen, triptans, and antihypertensive (ex. Propranolol) have been widely used in clinical trials to treat PTH.^[5]^ Antidepressant (ex. Tricyclic antidepressants) and anticonvulsants (ex. Pregabalin, Gabapentin) are used for post-trauma central pain.^[6,7]^ Also, some studies have reported the positive therapeutic effect of repetitive transcranial magnetic stimulation (rTMS) for PTH and central pain. Also, it is a potential treatment for some neuropsychiatric disorders.^[8–10]^

Mild TBI patients showed the microstructural damages in the long-distance White matter connection and it was well visualized using diffusion tensor imaging (DTI).^[11]^ DTI is a brain imaging to characterize the tree-dimensional diffusion of water as a function of spatial location.^[12]^ Spinothalamic tract (STT) is a major somatosensory tract and has been considered a plausible neural tract associated with central pain.^[13–15]^ The inferior longitudinal fasciculus tract (ILF), superior longitudinal fasciculus tract (SLF), and inferior fronto-occipital fasciculus (IFOF) tract play an important role in a wide range of cognitive and depression for mild traumatic brain injury.^[16,17]^

In previous studies, PTH, depression and central pain were shown through tractography derived from DTI and the therapeutic effect of rTMS was demonstrated by clinical improvement of symptoms.^[9,10,18]^ However, there has been no study specifically showing the effect of rTMS for post-trauma headache, depression and central pain through a damaged tractography derived from DTI. This study reports to show serial changes of related tract for PTH, depression and central pain by performing DTI conductions at 3 time points (before, during, and after rTMS) and to report a literature review.

## 2. Case description

This case study patient was a 51-year-old man with mild TBI who had no known chronic disease without any medication history before the accident. His head was injured by shaking effect of the car when crushing into guardrails. Right after the car accident, he had a loss of consciousness for <10 minutes, but had no neurologic sequelae such as motor deficits. After the car accident, headache, and depression had started and been treated at hospital despite of no specific findings on twice conventional brain magnetic resonance images. He complained headache focused on vertex area, depressive mood, cognitive impairment including concentration, working memory deficit, posterior neck, and right-side pain, and sleep disturbance. These symptoms persisted despite of medication and injection therapy in other hospitals for 13 months.

**Figure 1.**
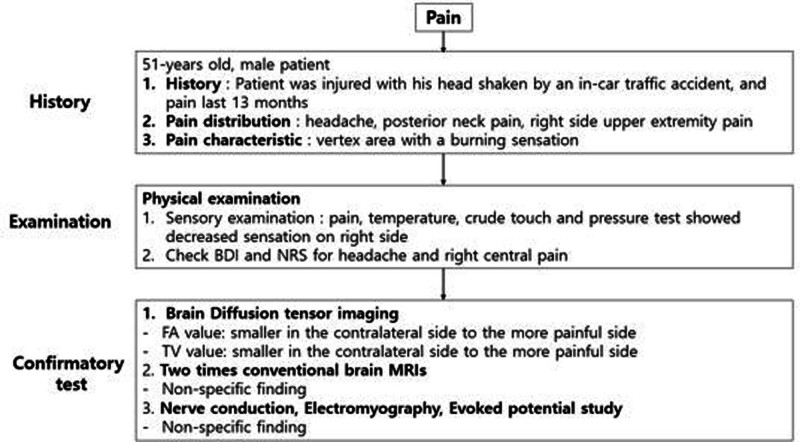
Flow chart of the grading system for neuropathic pain. MRI = magnetic resonance image, BDI = beck depression inventory, NRS = numeric rating scale.

He visited our hospital for further evaluation and treatment. His main complaints were headache and posterior neck pain commonly more on vertex area with a burning sensation and also accompanied central pain and right-side pain. Also, after the trauma he was easily irritated and depressed. We conducted physical sensory examination. When pain, temperature, crude touch, and pressure tests were performed, he showed decreased sensation on right side. There were no other abnormal findings on physical examination (Fig. 1).

We checked the headache and right central pain through a numeric rating scale (NRS), which is expressed as a score from 0 to 10, 0 means no pain, and 10 means maximum imaginable pain.^[19]^ Also, we checked depression intensity through a Beck depression inventory (BDI) score, which is evaluated from 0 to 63, 0 means no depressive mood, and 63 means maximum depressive mood.^[20]^ We performed the motor and sensory-evoked potential studies (Motor-evoked potential study, Sensory-evoked potential study), nerve conduction study, and electromyography to confirm the neural compromise. We conducted brain DTI 3 times to investigate the cause of pain; before, during, and after rTMS treatment. We acquired 49 contiguous slices parallel to the anterior commissure-posterior commissure line. Imaging parameters were as follows: acquisition time = approximately 3 minutes; acquisition matrix = 128 × 128; field of view = 220 mm × 220 mm; TR = 6600 ms; TE 95 ms; parallel acquisition factor GRAPPA = 2 bandwidth = 1562 Hz/Px; EPI factor = 128; b = 1000 s/mm^2^; slice gap = 0 mm; and a slice thickness = 3 mm (acquired isotropic voxel size = 0.9 mm × 0.9 mm × 3.0 mm).^[21]^ After DTI, fiber tracking was performed with fractional anisotropy (FA) threshold of 0.20 and a tract turning angle of 60^◦^ using the DTI studio software v.1.02 (CMRM, John Hopkins Medical Institute, Baltimore, MD, USA). We performed several DTI reconstructions by selecting 3 regions of interest from the brain magnetic resonance images axial slice; STT (the first target ROI was set the ventro-postero-lateral nucleus of the thalamus, the second ROI was both posterolateral to the inferior olivary nucleus and anterior to the inferior cerebellar peduncle in the medulla), SLF (the seed ROI was set the superior parietal lobule, the target ROI was the supplementary motor area along with the dorsal part of premotor area), ILF (the first target ROI was set the posterior border of anterior temporal lobe, the second target was around the occipital lobe), IFOF (the first target ROI was set the inferior frontal region, the second target was in the occipital region), uncinate from coronal slice (the first ROI was set the entire temporal lobe, the second target was the entire fiber projections in the frontal lobe on the posterior-most coronal slice).^[13,22–25]^ Subsequently, we selected fiber bundles that passed through the ROI. Then, the FA and tract volume (TV) of the reconstructed tracts were obtained, and the values of the left and right side were compared.^[14,26,27]^ The FA values represented the directionality of water diffusion and was quantified from 0 (completely isotropic diffusion) to 1 (completely anisotropic diffusion).^[26,28]^ The TV represents the number of voxels included in the neural tract.^[29,30]^

We applied rTMS as a treatment for central pain and depression. The following rTMS protocol was employed based on a previous study: high-frequency rTMS (10 Hz rTMS, 1500 pulses, 30 s cycles, 4 s on, 26 s off) applied to the left dorsolateral prefrontal cortex daily for 15 days, and intensity was 120% of motor threshold.^[31,32]^ rTMS was applied total 15 times; once a day and 5 times a week for 3 weeks.

**Figure 2.**
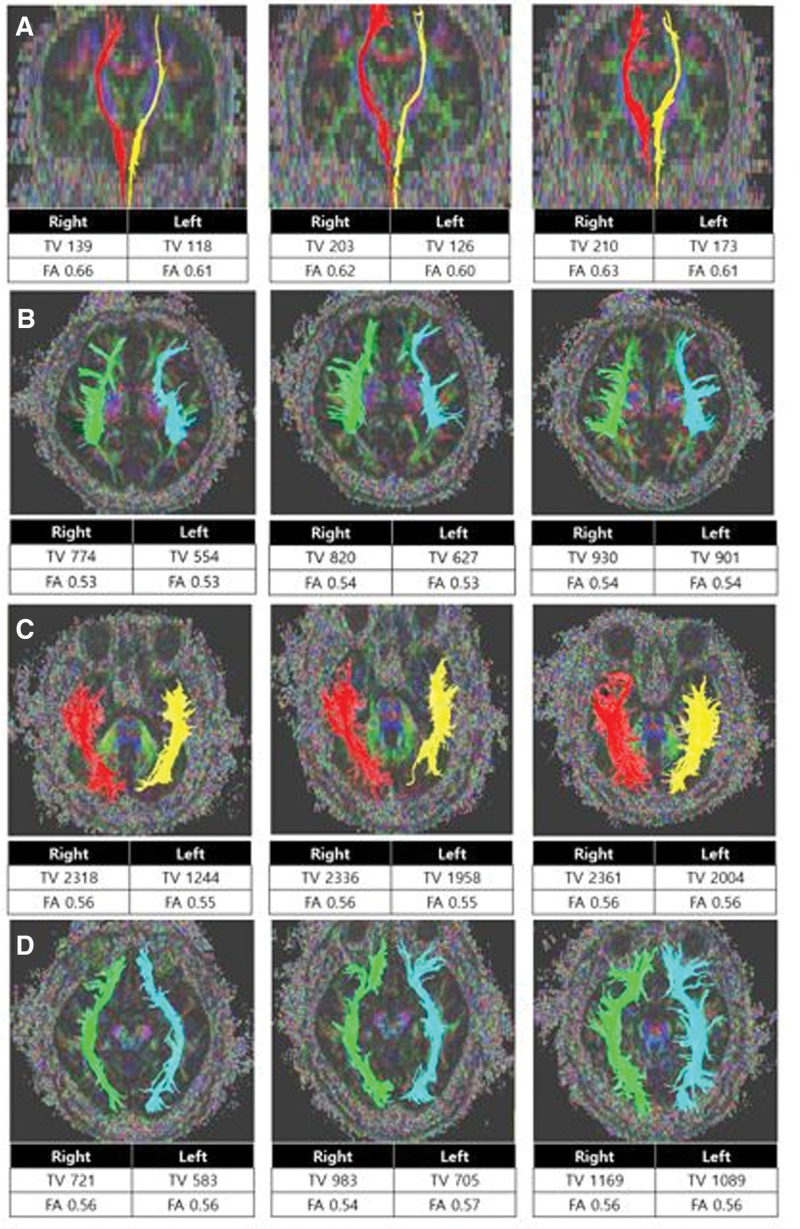
Tractography for the STT, SLF, ILF, and IFOF tracts in patient before, during, and after rTMS. (A) Tractography for STT before, during, and after rTMS. (B) tractography for SLF before, during, and after rTMS. (C) tractography for ILF before, during, and after rTMS. (D) tractography for IFOF before, during, and after rTMS. During and after rTMS, FA and TV values had increased compared to the values to the value before rTMS. rTMS = repetitive transcranial magnetic stimulation, FA = fractional anisotropy, TV = tract volume, SLF = superior longitudinal fasciculus tract, ILF = inferior longitudinal fasciculus tract, IFOF = inferior fronto-occipital fasciculus tract, STT = spinothalamic tract.

We checked headache, central pain NRS and BDI at 3 time points: admission, discharge and 1 month after discharge. NRS scores showed decrease from 6 to 3 and then to 3 (headache) and from 4 to 2 and then to 2 (right central pain). Also, BDI score showed improvement from 34 to 23 and then to 23 (Table 1). These scores were maintained after a year after discharge. Before rTMS, the FA and TV values of the contralateral STT, ILF, SLF, and IFOF on the more painful side were lower than on the other side. The FA and TV values during and after rTMS treatment were increased compared to the results before rTMS treatment (Fig. 2). The TV value in SLF changed from 774 to 930, in ILF from 2318 to 2361, in STT from 139 to 210, and in IFOF from 721 to 1169. The FA value in SLF changed from 0.53 to 0.54, in ILF from 0.56 to 0.56, in STT from 0.66 to 0.63, and in IFOF from 0.56 to 0.56. Since the patient complained of central pain on the right side, we evaluated the TV and FA values on the right side. During the rTMS treatment, there was no change in medications, other interventions and there was no adverse effect such as headache or dizziness.

**Table 1:** The NRS and BDI of patient before and after rTMS.

	Admission	Discharge	1 mo after discharge
NRS for headache	6	3	3
NRS for right central pain	4	2	2
BDI	34	23	23

BDI = beck depression inventory, NRS = numeric rating scale, rTMS = repetitive transcranial magnetic stimulation.

## 3. Discussion and conclusion

PTH is 1 of the most common complications of TBI and common clinical presentations are migraine or tension-type headache, posterior neck pain, and psychological symptoms.^[8]^ Also, post-traumatic central pain has a relatively late onset and it can show asymmetric neuropathic pain.^[33,34]^

Previous studies reported that rTMS was an effective treatment to relieve headache and central pain in post-traumatic brain injury patients.^[14,35]^ Both primary motor cortex and dorsolateral prefrontal cortex (DLPFC) show effective treatment results.^[36,37]^ The mechanisms of rTMS to the motor cortex for neuropathic pain can be explained by a reduction in pain-related thalamic hyperactivity, regional cerebral blood flow changes, activation of descending pathways, and restoration of intracortical inhibition.^[10,38,39]^ In other way, when stimulating DLPFC area, it occurs pain perception and regulation, with connections to other pain inhibitory regions such as anterior cingulate cortex and periaqueductal gray. DLPFC plays an important role in modulation of sensory, affective, and cognitive processing and inhibits a top-down or descending pain modulation pathway. rTMS above DLPFC area can be applied the 2000 pulses, 10 Hz, 4 sessions or 600 pulses, 10 Hz, 10 sessions.^[40–42]^ This patient’s main symptom was a headache and depression, so we applied rTMS on DLPFC area.

Chong, C.D., et al reported that there were significant DTI differences in anterior thalamic radiation, cingulum, inferior longitudinal fasciculi, uncinated fasciculus between persistent PTH patient and control group.^[43]^ Obermann, M., et al’s study showed that there was less gray matter density in anterior cingulate cortex and dorsolateral prefrontal cortex in persistent PTH in comparison with healthy group.^[44]^ If there were neither many comparison groups nor normal data, central pain was approached through side-to-side comparison of STT.^[14,45]^ However, previous rTMS treatment studies mostly compared DTI between normal and patients or showed difference between left and right tracts. There has been no report that objectively confirmed the effect of rTMS on DTI-based STT metrics measurement of FA and TV of FA and volume changes in White matter related to pain and depression from serial DTI. The STT was considered the most plausible neural tract responsible for the pathogenesis of central poststroke pain.^[15]^ The SLF is an important bundle of association fibers connecting the parietal, occipital and temporal lobes; core process such as attention, memory, emotions, and language. The ILF is a direct connection tract from the occipital to temporal lobe, running laterally and inferiorly above radiation fibers. The IFOF runs medially and above the optic pathway and it connects the occipital, posterior temporal and the orbitofrontal areas. These tracts play important roles in headache and depression so we selected these tracts to see the improvement of these symptoms. Most of the presenting studies on the effectiveness of rTMS have been showed as symptomatic improvement in patients.

Our patient had complained PTH, depression and right-sided central pain. After 15th rTMS treatment, he showed decrease in NRS both for headache from 6 to 3 and for right-sided central pain from 4 to 2. Also, BDI score showed improvement from 34 to 23. In addition, we obtained DTI images at 3 time points (before, during-day of rTMS 6th stimulation and after-day of rTMS 15th stimulation) to show several DTI tracts; SLF and ILF related to headache, STT which is related to central pain, and IFOF related to depression. As a result, we found that both FA and TV values had increased as treatment progress. This case is meaningful in that not only pain and depression were improved after the rTMS but also serial changes in White matter were observed in DTI serial changes. This findings could be an objective evidence to explain a restoration of intracortical inhibition due to activation of impaired White matter related to headache, pain and depression through rTMS treatment.^[38,46]^

This case report has the following limitations. It is difficult to evaluate the duration of therapeutic effect of rTMS treatment 1 year after the final treatment and lost the patient’s voluntary outpatient follow up. In addition, as it is only a case study, a randomized controlled trial is needed to prove a clearer effect of the treatment. It is difficult to enroll patients with similar symptoms and its cost burden is too high to take serial DTI images. Later, case series studies or randomized controlled trials will be needed in the future to more clearly verify the results of this study.

We report a rare case of changes in DTI-based metrics of White matter related to pain and depression before, during, and after rTMS, as well as the efficacy of rTMS in patients with mild TBI with headache and depressed mood and central pain.

## Acknowledgments

The authors extend their appreciation to all members of the Department of Physical Medicine & Rehabilitation at Jeonbuk National University Hospital.

## Author contributions

**Conceptualization:** Young-Ji Yun, Gi-Wook Kim.

**Formal analysis:** Young-Ji Yun, Gi-Wook Kim.

**Investigation:** Young-Ji Yun, Gi-Wook Kim.

**Methodology:** Young-Ji Yun, Gi-Wook Kim.

**Supervision:** Gi-Wook Kim.

**Writing – original draft:** Young-Ji Yun.

**Writing – review & editing:** Gi-Wook Kim.
